# Methodological and reporting concerns regarding “Melatonin improves fertilization rate in assisted reproduction: systematic review and meta-analysis”

**DOI:** 10.1016/j.clinsp.2026.101148

**Published:** 2026-10-01

**Authors:** Sedighe Esmaeilzadeh, Parvaneh Manabí

**Affiliations:** Infertility and Reproductive Health Research Center, Health Research Institute, Babol University of Medical Sciences, Babol, Iran

Dear Editor,

We read with great interest the systematic review and meta-analysis by de Arruda Veiga et al. evaluating melatonin supplementation in assisted reproductive technology outcomes.^1^ While the topic is timely and clinically relevant, we have several concerns regarding the methodology, study selection, and reporting that warrant caution in interpreting the findings.

First, the literature search was limited primarily to PubMed and SciELO, without mention of Embase, Web of Science, Scopus, Cochrane Central, gray literature, or trial registries. The full search strategy was not provided, limiting reproducibility and adherence to PRISMA standards.

Second, the eligibility criteria were insufficiently defined. Although the authors stated that they used the PICO framework, the inclusion and exclusion criteria remain ambiguous. Specifically, the eligible study designs were not clearly specified. While the authors reported excluding “animal studies” and “review articles”, they did not clarify whether non-randomized studies, quasi-experimental studies, case-control studies, retrospective analyses, or study protocols were eligible for inclusion. Furthermore, the intervention and comparator definitions across study arms were not adequately described.

Third, major discrepancies were identified in study design classification and referencing. Table 1 of de Arruda Veiga et al. was entitled “Exogenous melatonin in previous clinical studies”; however, several included studies do not meet the definition of clinical trials and instead evaluated endogenous melatonin levels or observational associations with IVF outcomes. For example, Fernando et al.^2^ represents a study protocol rather than a completed clinical trial with outcome data. Tong et al.^3^ and Zheng et al.^4^ primarily investigated endogenous follicular fluid melatonin concentrations and their association with reproductive outcomes rather than evaluating exogenous melatonin supplementation. Similarly, Li et al.^5^ was a case-control study, while Ma et al.^6^ was a retrospective multivariate logistic regression analysis rather than an interventional trial.

Fourth, some included studies evaluated combination therapies rather than melatonin monotherapy, including melatonin combined with myo-inositol and/or vitamin D. Such co-interventions may confound the isolated effect attributable to melatonin and contribute substantially to clinical heterogeneity.

Fifth, substantial statistical heterogeneity was reported across multiple analyses (fertilization rate: *I*^2^=88%; MII oocytes: *I*^2^=87%; follicle count: *I*^2^=85%; BMI: *I*^2^=77%). Despite this, no subgroup analyses, sensitivity analyses, or meta-regression approaches were presented to explore potential sources of heterogeneity or assess the robustness of the pooled estimates. High unexplained heterogeneity substantially weakens the reliability and interpretability of the meta-analytic findings.

Furthermore, several discrepancies were identified between the citations presented in Table 1 of de Arruda Veiga et al. and those listed in the final bibliography. For example, “Unfer et al.^7^ in Table 1 of de Arruda Veiga et al. corresponds to Reference 26 in the bibliography rather than Reference 19. Additional inconsistencies were observed in the citation numbering of the Zheng et al. studies, while References 9 and 29 appear to duplicate the same Li et al. (2019) publication. Similarly, References 10 and 34 appear to duplicate the same Zheng et al. (2023) article. These referencing inconsistencies and study design misclassifications complicate verification of the included evidence and may raise concerns regarding the accuracy of study selection and data extraction.

Given these methodological and reporting concerns, the conclusions of the review should be interpreted with caution. Greater transparency in study selection, clearer classification of study designs, and additional analyses exploring heterogeneity would strengthen the reliability and clinical applicability of the findings.

## Author’s contribution

Both authors contributed equally to the conception, critical analysis, and writing of this commentary. Both authors reviewed and approved the final version for submission.

### Ethics approval and consent to participate

Not applicable.

## Funding

This research did not receive any specific grant from funding agencies in the public, commercial, or not-for-profit sectors.

## **Data availability**

Not applicable.

## Declaration of competing interest

The authors declare no conflicts of interest.
